# Proteinuria as a Nascent Predictor of Frailty Among People With Metabolic Syndrome: A Retrospective Observational Study

**DOI:** 10.3389/fpubh.2022.847533

**Published:** 2022-03-10

**Authors:** Pi-Kai Chang, Yuan-Ping Chao, Li-Wei Wu

**Affiliations:** ^1^Division of Colon and Rectal Surgery, Department of Surgery, Tri-Service General Hospital and School of Medicine, National Defense Medical Center, Taipei, Taiwan; ^2^Graduate Institute of Medical Sciences, National Defense Medical Center, Taipei, Taiwan; ^3^Division of Family Medicine, Department of Family and Community Medicine, Tri-Service General Hospital, School of Medicine, National Defense Medical Center, Taipei, Taiwan; ^4^Division of Geriatric Medicine, Department of Family and Community Medicine, Tri-Service General Hospital; and School of Medicine, National Defense Medical Center, Taipei, Taiwan

**Keywords:** proteinuria, albumin to creatinine ratio, metabolic sybdrome, fraility, National Health and Nutrition Examination Survey

## Abstract

Frailty is a commonly occurring geriatric condition that increases the risk of adverse health outcomes. The factors and predictors behind frailty are not yet well understood. A better understanding of these factors can enable prevention of frailty in elderly patients. The objective of this study was to determine the association between proteinuria and frailty in US individuals with metabolic syndrome (MetS). Data from the National Health and Nutrition Examination Survey III (NHANES III, 1988–1994) conducted by the National Center for Health Statistics of the Centers for Disease Control and Prevention. This is a cross-sectional study, and proteinuria and frailty were measured only once at enrollment. The study included 2,272 participants with MetS aged 40–90 years from the NHANES III. The participants underwent assessments to evaluate frailty and frailty components (low body weight, weakness, exhaustion, low physical activity, and slow walking). Proteinuria was represented as albumin-to-creatinine ratio (ACR) (mg/g) and divided into tertiles: T1-normal range (ACR <30 mg/g), T2-microalbuminuria (ACR 30–299 mg/g), and T3-macroalbuminuria (ACR ≥ 300 mg/g). We applied multiple logistic regression to determine the odds ratios (ORs) of frailty for T2 vs. T1 and T3 vs. T1 in both sexes. In the adjusted analysis for male participants, the ORs of frailty for T2 and T3 vs. T1 were 3.106 (95% confidence interval [CI] = 1.078–8.948, *P* = 0.036) and 14.428 (95% CI = 4.231–49.193, *P* < 0.001), respectively. For female participants, the ORs of frailty for T2 and T3 vs. T1 were 1.811 (95% CI = 1.071–3.063, *P* = 0.027) and 2.926 (95% CI = 1.202–7.124, *P* = 0.018), respectively. The positive association between T2 and T3 vs. T1, and frailty were statistically significant. The trends of higher likelihood of every frailty component were also statistically significant across increasing tertiles of proteinuria after multiple levels of adjustment for covariates (*P* < 0.05). Increased proteinuria levels were positively associated with frailty and each frailty component. Proteinuria might be a useful maker for frailty in individuals with MetS.

## Introduction

Metabolic syndrome (MetS) is defined as a collection of cardiovascular risk factors, including elevated levels of triglycerides, low concentrations of high-density lipoprotein cholesterol, impaired fasting glucose, central obesity, and elevated blood pressure (1). The prevalence rate of MetS is high and is increasing worldwide (2). MetS is a risk factor for developing diabetes and cardiovascular disease and raises the possibility of all-cause and cardiovascular mortality in aging individuals (3).

The ever-increasing average age of the population has increased the interest of researchers in frailty. Frailty is a widely prevalent geriatric syndrome that reflects a state of decreased physiological reserve and increased vulnerability to stressors. Frail, older adults are at an increased risk of adverse health outcomes such as institutionalization, comorbidity, and mortality. Developing a better understanding of indicators that can be used to identify high-risk individuals is a major step toward preventing frailty. A previous study suggested that frailty is associated with chronic kidney diseases (CKDs), and albuminuria is an early indicator of diabetic nephropathy (4–6). Although CKDs have been extensively documented as a crucial factor in frailty, data examining the associations of early indicators of CKDs, such as albuminuria, with frailty are relatively sparse. Albuminuria is associated with frailty among community-dwelling middle-aged and older people (7). Given the aging population, it may be beneficial to use laboratory data for the early screening of frailty. This study investigated the correlation between different levels of proteinuria, frailty, and each frailty component (slowing walking, weakness, exhaustion, low physical activity, and low body weight) among people with MetS. Representative samples were retrieved from the U.S. National Health and Nutrition Examination Survey III (NHANES III, 1988–1994).

## Materials and Methods

### Data Source and Participants

**Figure 1:**
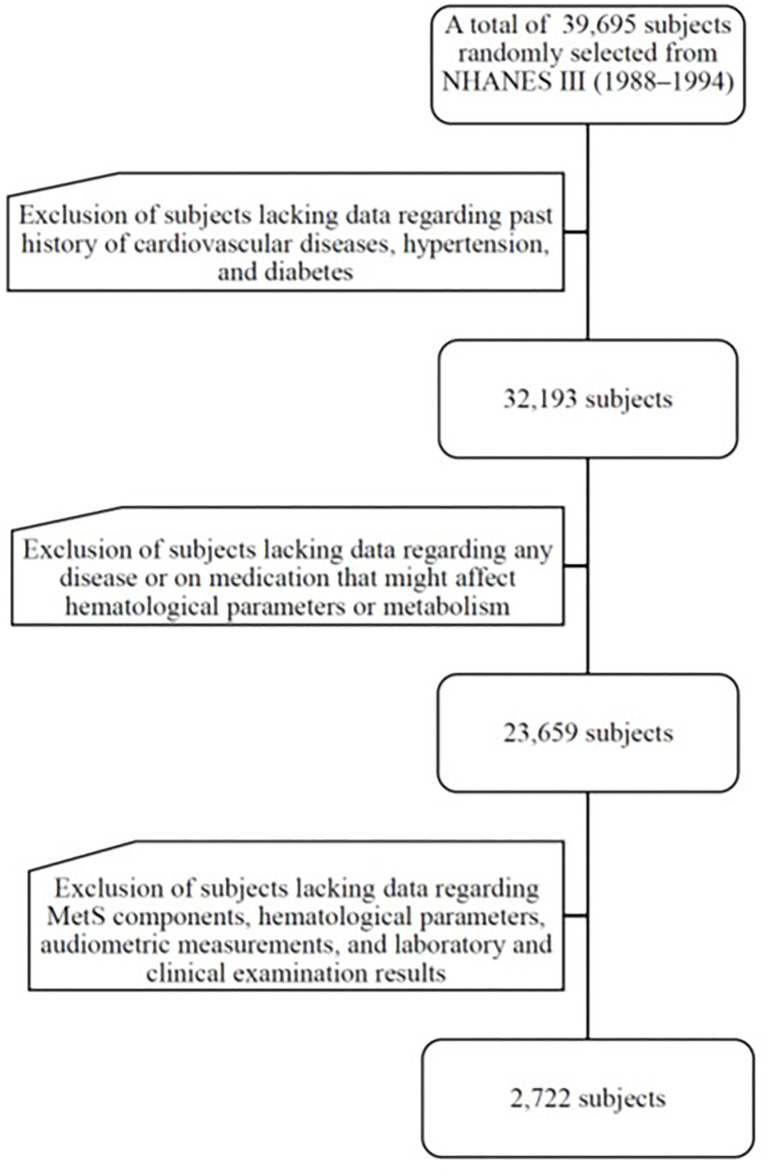
Flow diagram of the selection of participants.

The data were from NHANES III (1988–1994), a nationwide probability sample of 39,695 persons aged 2 months and older. The National Center for Health Statistics (NCHS) of the Centers for Disease Control and Prevention (CDC) introduced this cross-sectional survey to assess the health and nutritional status of Non-institutionalized U.S. residents. The survey included all ethnicities, such as Non-Hispanic white, Non-Hispanic black, Mexican American and others of community-dwelling populations of the US. The retrieved data were used with informed consent, and the participants were examined in a mobile center. The institutional review board (IRB) exempted the protocol from a formal review owing to the anonymous nature of the data. The survey was executed in accordance with the Declaration of Helsinki and was based on a complex, multistage, stratified, clustered probability design. Detailed operations manuals, consent documents, and brochures of NHANES III are available online at http://www.cdc.gov/nchs/nhanes.htm. The study flowchart is shown in Figure 1. To eliminate the influence from possible confounding factors, we've determined exclusion criteria as follows: subjects lacking data on past medical history of cardiovascular (CV) diseases, hypertension, type 2 diabetes mellitus (DM) (*n* = 7,502); subjects lacking data on any disease or taking antidiabetic agents, antihypertensive medications, and lipid-lowering drugs that might affect biochemical parameters or lipid metabolism (*n* = 8,534). In addition, subjects lacking data on MetS components, lipid profile, and the results of laboratory as well as clinical examinations or lost follow-up (*n* = 21,387) were excluded. This study enrolled 2,272 participants aged 40–90 years.

### Measurement: Proteinuria

A casual urine specimen was collected from each participant in sterile containers. The samples were stored in frozen conditions (−20°C). Dipstick methods are not effective at detecting low levels of urinary albumin; therefore, the study employed a solid-phase fluorescent immunoassay for the measurement. Urine creatinine was analyzed with the Jaffe reaction using a Beckman Synchron AS/ASTRA analyzer (Beckman Coulter, Fullerton, California) in which creatinine reacted with picrate in an alkaline solution to form a red creatinine–picrate complex. To measure urinary albumin excretion, the albumin-to-creatinine ratio (ACR) was calculated by dividing the urinary albumin value by the urinary creatinine concentration (8, 9). Proteinuria was divided into three tertiles. Those with ACR <30 mg/g were referred to as the normal range group (tertile 1, T1). Microalbuminuria (tertile 2, T2) and macroalbuminuria (tertile 3, T3) were defined as ACR of 30–299 mg/g and ≥ 300 mg/g, respectively.

### Measurement: Frailty

A validated five-item frailty phenotype score proposed by Fried et al. was adapted (10). The frailty phenotype consists of the following five items:

1. Slow walking: defined as the slowest quintile adjusted for sex in a timed eight-foot walk.
2. Weakness: defined by “some difficulty, much difficulty,” or “unable to do” when asked how much difficulty they have “lifting or carrying something as heavy as 10 pounds”.
3. Exhaustion: defined by “some difficulty, much difficulty,” or “unable to do” when asked how much difficulty they have “walking from one room to the other on the same level”.
4. Low physical activity: defined as present if participants answered “less active” when asked “Compared with most (men/women) your age, would you say that you are more active, less active or about the same?”
5. Low body weight: defined by body mass index (BMI) ≤ 18.5 kg/m^2^.

In this study, individuals were considered frail if they exhibited three or more of the mentioned items.

### Measurement: Correlation Between Proteinuria and Frailty

Although low-grade albuminuria plays a role in frailty and may share common pathological mechanisms related to CV diseases (7), the potential effect of increasing levels of proteinuria on frailty and each component of frailty remains unknown. Thus, we clarified the relationship between different degrees of proteinuria (normal range, microalbuminuria, macroalbuminuria), frailty, and frailty components in the study.

### Measurement: Risk Variables

Self-report history was obtained for the following variables by asking the question “Has a doctor ever told you that you have (disease state) congestive heart failure (CHF), skin cancer, any other cancer, stroke, asthma, or DM?”. The interviews were conducted by trained personnel. Questions were directed to the respondent or, if necessary, to their proxy. Physical activity was determined by asking, “Are you active compared to men/women your age?” Smokers were identified by asking, “Do you smoke cigarettes?” while “ever smokers” included subjects answering “yes” to “Have you ever smoked at least 100 cigarettes in your lifetime?”

Metabolic variables were obtained from blood samples. Plasma glucose was measured from fasting blood samples (fasted for 6 h or more) using the hexokinase enzymatic method. Serum total cholesterol (TC), serum total triglycerides (TG), serum high-density lipoprotein (HDL), and serum low-density lipoprotein (LDL) were measured using a Hitachi 704 analyzer (Roche Diagnostics, Indianapolis, IN, USA). Serum C-reactive protein (CRP) concentration was measured by latex-enhanced nephelometry with a Behring Nephelometer Analyzer System (Behring Diagnostics Inc.). This study utilized the Hitachi 737 Analyzer to measure other biochemical profiles, such as serum uric acid (UA), serum total bilirubin, aspartate aminotransferase (AST), alanine aminotransferase (ALT), serum creatinine, and serum total protein. Urinary creatinine and urinary albumin levels were measured using the Synchron AS/ASTRA Clinical Analyzer and fluorescent immunoassay, respectively. Age, BMI, systolic blood pressure (SBP), diastolic blood pressure (DBP), and blood and urinary samples were listed as continuous variables. The database had already been approved by the CDC and appropriate permissions and ethical clearance were obtained.

### Statistical Analysis

All statistical analyses were conducted using the Predictive Analytics Suite Workstation Statistics (SPSS Inc., Chicago, IL, USA). The NHANES III is a database with complex designs. It was inappropriate to perform statistical analyses with the assumption of a simple random sample providing incorrect variance estimates. “Complex Sampling” was used to incorporate sample weights and adjusted for strata of the complex sample design. Basic statistics were used to describe the characteristics of the study participants. Continuous variables are presented as mean ± standard deviation (SD), while categorical variables are presented as counts and percentages (%). ANOVA was used for analyzing continuous variables, and the Chi-square test was used for categorical variables.

We used multiple logistic regression to determine the odds ratios (ORs) of frailty for increasing proteinuria levels. The participants in the lowest group being used as the reference group. The OR for frailty was obtained using multiple logistic regression by comparing each participant in the upper two tertiles of the proteinuria levels to those in the lowest tertile. For covariate adjustment, we used an extended-model approach: Model 1 was unadjusted by other variables; Model 2 was an adjusted Model 1, and was adjusted by the age, ethnicity, and BMI variables; Model 3 was an adjusted Model 2, and was adjusted by the SBP, serum fasting glucose, serum TG, and serum creatinine variables; Model 4 was an adjusted Model 3, and was adjusted by the history of congestive heart failure, stroke, diabetes mellitus, smoker, and physical activity variables. Trend tests were assessed by treating the tertiles of proteinuria levels from T1 to T3 as a continuous variable to observe the associations across increasing tertiles of proteinuria levels and OR of frailty.

## Results

### Study Sample Characteristics

The study consisted of 2,272 adults aged 40–90 years whose frailty measures and laboratory examinations were available. The baseline characteristics of the subjects are presented in Table 1. The mean age was 72.07 years in women and 71.73 years in men, and 1,010 subjects (44.5%) were male. There were statistically significant differences in age, serum UA, serum creatinine, urinary albumin, history of CHF, DM, and physical activity between the frail and Non-frail groups in both sexes. There were also statistically significant differences in BMI, serum TG, serum glucose, serum total protein, and history of stroke in women, and DBP, serum HDL, and urinary creatinine in men between the frail and Non-frail groups.

**Table 1:** Metabolic syndrome participant characteristics by gender, frailty, and Non-frailty.

**Variables**	**Male group** ***N*** **=** **1,010**				**Female group** ***N*** **=** **1,262**			
	**Non-frailty *N* = 971**	**Frailty *N* = 39**	**Total *N* = 1,010**	***P* *-*value**	**Non-frailty *N* = 1,152**	**Frailty *N* = 110**	**Total *N* = 1,262**	***P* *-*value**
**Continuous variables**								
BMI (kg/m^2^), mean (SD)	28.27 (4.10)	28.05 (4.99)	28.26 (4.14)	0.754	28.79 (5.48)	32.03 (7.00)	29.07 (5.70)	<0.001
Age (years), mean (SD)	71.54 (7.92)	76.38 (7.47)	71.73 (7.96)	<0.001	71.89 (7.97)	74.02 (8.24)	72.07 (8.01)	0.008
SBP (mmHg), mean (SD)	146.78 (20.89)	146.84 (24.08)	146.79 (21.00)	0.987	148.74 (22.45)	145.98 (21.39)	148.50 (22.36)	0.223
DBP (mmHg), mean (SD)	77.14 (12.25)	72.79 (19.11)	76.97 (12.60)	0.037	71.55 (14.72)	70.64 (13.34)	71.47 (14.68)	0.541
Serum TG (mg/dL)	163.23 (122.63)	169.90 (141.57)	163.49 (123.34)	0.741	165.70 (103.08)	220.55 (172.98)	170.48 (111.92)	<0.001
Serum cholesterol (mg/dL), mean (SD)	209.68 (43.00)	207.67 (45.73)	209.60 (43.08)	0.775	231.85 (44.24)	230.39 (47.43)	231.72 (44.51)	0.743
Serum LDL-cholesterol (mg/dL), mean (SD)	135.66 (35.72)	134.54 (30.26)	135.63 (35.56)	0.911	146.17 (40.03)	137.73 (34.69)	145.55 (39.69)	0.196
Serum HDL-cholesterol (mg/dL), mean (SD)	43.14 (13.29)	47.82 (18.48)	43.32 (13.55)	0.034	53.02 (16.98)	52.40 (17.54)	52.96 (17.03)	0.718
Serum CRP (mg/dL), mean (SD)	0.59 (0.96)	0.70 (0.61)	0.59 (0.94)	0.470	0.63 (0.97)	0.76 (0.89)	0.64 (0.97)	0.202
Serum total bilirubin (umol/L), mean (SD)	0.67 (0.35)	0.61 (0.23)	0.67 (0.34)	0.317	0.52 (0.24)	0.50 (0.31)	0.52 (0.25)	0.639
Serum UA (mg/dL), mean (SD)	6.24 (1.50)	6.81 (1.72)	6.26 (1.51)	0.021	5.45 (1.45)	5.89 (1.71)	5.49 (1.48)	0.003
Serum glucose (mg/dL), mean (SD)	121.46 (49.40)	126.74 (41.99)	121.66 (49.13)	0.510	117.11 (47.87)	145.04 (78.29)	119.55 (51.81)	<0.001
Serum total protein (g/dL), mean (SD)	7.38 (0.48)	7.44 (0.42)	7.38 (0.47)	0.391	7.32 (0.49)	7.46 (0.56)	7.33 (0.50)	0.005
AST (U/L), mean (SD)	22.34 (14.14)	21.79 (10.03)	22.32 (14.00)	0.812	21.21 (11.47)	21.07 (9.48)	21.20 (11.31)	0.902
ALT (U/L), mean (SD)	15.93 (12.71)	14.36 (9.25)	15.87 (12.60)	0.446	14.21 (9.40)	13.82 (8.51)	14.18 (9.32)	0.671
Serum creatinine (mg/dL), mean (SD)	1.33 (0.65)	1.56 (0.66)	1.34 (0.65)	0.030	1.09 (0.435)	1.21 (0.970)	1.10 (0.505)	0.019
Urinary albumin (ug/mL), mean (SD)	113.59 (495.40)	319.79 (649.43)	120.49 (502.27)	0.020	73.01 (561.04)	233.95 (903.47)	86.51 (598.49)	0.009
Urinary creatinine (mg/dL), mean (SD)	127.49 (66.60)	101.71 (49.32)	126.63 (66.24)	0.028	92.05 (60.69)	88.47 (51.24)	91.75 (59.94)	0.563
**Categorical variables**								
Non-hispanic white *N* (%)	200 (20.6)	6 (15.4)	206 (20.4)	0.375	224 (19.4)	35 (31.8)	259 (20.5)	0.006
Congestive heart failure, *N* (%)	99 (10.2)	10 (25.6)	109 (10.8)	0.025	77 (6.7)	17 (15.5)	94 (7.4)	0.003
Stroke, *N* (%)	68 (7.0)	6 (15.4)	74 (7.3)	0.267	71 (6.2)	22 (20.0)	93 (7.4)	<0.001
Asthma, *N* (%)	52 (5.4)	1 (2.6)	53 (5.2)	0.730	82 (7.1)	11 (10.0)	93 (7.4)	0.269
Skin cancer, *N* (%)	119 (12.3)	5 (12.8)	124 (12.3)	0.916	87 (7.6)	6 (5.5)	93 (7.4)	0.688
Other cancer, *N* (%)	70 (7.2)	4 (10.3)	74 (7.3)	0.759	82 (7.1)	8 (7.3)	90 (7.1)	0.952
Diabetes mellitus, *N* (%)	185 (19.1)	14 (35.9)	199 (19.7)	0.034	243 (21.1)	44 (40.0)	287 (22.7)	<0.001
Smoker, *N* (%)	350 (36.0)	15 (38.5)	365 (36.1)	0.758	7 (0.6)	0 (0)	7 (0.6)	0.412
Physical activity, n (%)				<0.001				<0.001
Ideal, *N* (%)	351 (36.1)	13 (33.3)	364 (36.0)		324 (28.1)	16 (14.5)	340 (26.9)	
Intermediate, *N* (%)	451 (46.4)	5 (12.8)	456 (45.1)		465 (40.4)	17 (15.5)	482 (38.2)	
None, *N* (%)	169 (17.4)	21 (53.8)	190 (18.8)		363 (31.5)	77 (70.0)	440 (34.9)	

*N, number; SD, standard deviation; BMI, body mass index; SBP, systolic blood pressure; DBP, diastolic blood pressure; serum TG, serum triglycerides; LDL, low-density lipoprotein; HDL, high-density lipoprotein; serum CRP, serum C-reactive protein; serum UA, serum uric acid; AST, aspartate aminotransferase; ALT, alanine aminotransferase*.

### Preliminary Analysis

To determine the relationship between proteinuria and frailty, a multivariable-adjusted logistic regression analysis was performed to demonstrate the effect of proteinuria. Table 2 shows the significant positive associations between proteinuria tertiles and frailty. In the unadjusted analysis for male participants, the ORs of frailty for T2/T1 and T3/T1 were 3.152 (95% confidence interval [CI] = 1.120–8.870, *P* = 0.030) and 18.620 (95% CI = 6.980–49.674, *P* < 0.001), respectively. For female participants, the ORs of frailty for T2/T1 and T3/T1 were 2.203 (95% CI = 1.344–3.611, *P* = 0.002) and 5.296 (95% CI = 2.482–11.300, *P* < 0.001), respectively. After additionally adjusting for other covariates in Models 2–4, the positive association between T2/T1, T3/T1, and frailty remained essentially unchanged in both sexes. In the male group, the ORs of frailty for T2/T1 were 2.891 (95% CI = 1.035–8.297, *P* = 0.041), 3.217 (95% CI = 1.065–8.510, *P* = 0.038), and 3.106 (95% CI = 1.078–8.948, *P* = 0.036) from Models 2 to 4, respectively. In the female group, the ORs of frailty for T2/T1 were 1.872 (95% CI = 1.151–3.742, *P* = 0.013), 1.928 (95% CI = 1.163–3.191, *P* = 0.011), and 1.811 (95% CI = 1.071–3.063, *P* = 0.027) from Models 2 to 4, respectively. The ORs of frailty for T3/T1 were higher than those for T2/T1 in both sexes. For the male participants, the ORs of frailty for T3/T1 were 15.517 (95% CI = 4.926–48.940, *P* < 0.001), 14.845 (95% CI = 4.703–47.887, *P* < 0.001), and 14.428 (95% CI = 4.231–49.193, *P* < 0.001) from Models 2 to 4, respectively. For the female participants, the ORs of frailty for T3/T1 were 4.454 (95% CI = 2.186–9.673, *P* < 0.001), 3.108 (95% CI = 1.247–7.253, *P* = 0.014), and 2.926 (95% CI = 1.202–7.12, *P* = 0.018) from Models 2 to 4, respectively.

**Table 2:** Association between the frailty and proteinuria in male and female participants with metabolic syndrome.

**Models^**a**^**	**Tertiles**	**Male group**			**Female group**		
		**Odds ratio^**b**^ (95% CI)**	***P* Value**	***P* for Trend**	**Odds ratio^**b**^ (95% CI)**	***P* Value**	***P* for Trend**
Model 1	T2 vs. T1	3.152 (1.120, 8.870)	0.030	<0.001	2.203 (1.344, 3.611)	0.002	<0.001
	T3 vs. T1	18.620 (6.980, 49.674)	<0.001		5.296 (2.482, 11.300)	<0.001	
Model 2	T2 vs. T1	2.891 (1.035, 8.297)	0.041	<0.001	1.872 (1.151, 3.742)	0.013	<0.001
	T3 vs. T1	15.517 (4.926, 48.940)	<0.001		4.454 (2.186, 9.673)	<0.001	
Model 3	T2 vs. T1	3.217 (1.065, 8.510)	0.038	<0.001	1.928 (1.163, 3.191)	0.011	<0.001
	T3 vs. T1	14.845 (4.703, 47.887)	<0.001		3.108 (1.247, 7.253)	0.014	
Model 4	T2 vs. T1	3.106 (1.078, 8.948)	0.036	<0.001	1.811 (1.071, 3.063)	0.027	<0.001
	T3 vs. T1	14.428 (4.231, 49.193)	<0.001		2.926 (1.202, 7.124)	0.018	

*T1, normal range (ACR <30 mg/g); T2, microalbuminuria (ACR 30–299 mg/g); and T3, macroalbuminuria (ACR ≥ 300 mg/g); ACR: albumin-to-creatinine ratio*.

^a^Adjusted covariates:

Model 1 = Unadjusted.

Model 2 = Model 1 + age, ethnicity, body mass index (BMI).

Model 3 = Model 2 + systolic blood pressure (SBP), serum fasting glucose, serum TG, serum creatinine.

Model 4 = Model 3 + history of congestive heart failure, stroke, diabetes mellitus, smoker, physical activity.

^b^Odds ratios were interpreted as change of frailty for each increase in different proteinuria levels.

### Association Between Proteinuria and the Components of Frailty

Prominent frailty measures included the frailty phenotype (10) and the frailty index (11). This investigation adapted frailty phenotype as a measurement. Table 3 shows that proteinuria was significantly positively associated with each frailty component. Participants in the higher tertiles of proteinuria tended to have higher ORs for each frailty component. The trends of higher likelihood of all frailty components were statistically significant across increasing tertiles of proteinuria after additionally adjusting for other covariates from Models 1 to 4 (*P* < 0.05 for all trends).

**Table 3:** Association between the frailty components and proteinuria in male and female participants with metabolic syndrome.

**Models^**a**^**	**Tertiles**	**Male group**			**Female group**		
		**Odds ratio^**b**^ (95% CI)**	***P* Value**	***P* for Trend**	**Odds ratio^**b**^ (95% CI)**	***P* Value**	***P* for Trend**
**Frailty (Slow walking)**							
Model 1	T2 vs. T1	2.049 (1.282, 3.275)	0.003	<0.001	2.026 (1.474, 2.785)	<0.001	<0.001
	T3 vs. T1	5.423 (3.046, 9.654)	<0.001		6.133 (3.938, 9.553)	<0.001	
Model 2	T2 vs. T1	1.857 (1.187, 3.005)	0.006	<0.001	1.721 (1.257, 2.319)	0.001	<0.001
	T3 vs. T1	4.587 (2.714, 8.107)	<0.001		6.001 (4.003, 9.719)	<0.001	
Model 3	T2 vs. T1	1.918 (1.286, 3.180)	0.008	<0.001	1.778 (1.301, 2.517)	0.001	<0.001
	T3 vs. T1	4.514 (2.245, 8.359)	<0.001		4.457 (2.488, 7.963)	<0.001	
Model 4	T2 vs. T1	1.820 (1.125, 2.943)	0.015	<0.001	1.578 (1.133, 2.198)	0.007	<0.001
	T3 vs. T1	4.337 (2.188, 8.597)	<0.001		4.162 (2.471, 7.012)	<0.001	
**Frailty (Weakness)**							
Model 1	T2 vs. T1	1.942 (1.260, 2.993)	0.003	<0.001	1.964 (1.565, 2.465)	<0.001	<0.001
	T3 vs. T1	6.370 (3.961, 10.995)	<0.001		3.209 (2.127, 4.841)	<0.001	
Model 2	T2 vs. T1	1.465 (0.994, 2.268)	0.079	<0.001	1.747 (1.388, 2.194)	<0.001	<0.001
	T3 vs. T1	4.518 (2.637, 8.110)	<0.001		3.121 (2.024, 4.783)	<0.001	
Model 3	T2 vs. T1	1.453 (0.912, 2.253)	0.099	<0.001	1.826 (1.431, 2.276)	<0.001	<0.001
	T3 vs. T1	4.220 (2.263, 7.886)	<0.001		2.456 (1.593, 3.812)	<0.001	
Model 4	T2 vs. T1	1.398 (0.900, 2.171)	0.136	<0.001	1.642 (1.297, 2.080)	<0.001	<0.001
	T3 vs. T1	4.252 (2.267, 7.975)	<0.001		2.306 (1.467, 3.623)	<0.001	
**Frailty (Exhaustion)**							
Model 1	T2 vs. T1	1.895 (0.928, 3.871)	0.079	<0.001	2.035 (1.276, 3.247)	0.003	<0.001
	T3 vs. T1	7.850 (3.679, 16.752)	<0.001		3.553 (1.624, 7.771)	0.001	
Model 2	T2 vs. T1	1.424 (0.695, 2.978)	0.412	<0.001	1.627 (1.019, 2.613)	0.044	<0.001
	T3 vs. T1	5.277 (2.457, 11.335)	<0.001		2.869 (1.299, 6.338)	0.009	
Model 3	T2 vs. T1	1.462 (0.767, 3.122)	0.335	<0.001	1.657 (1.215, 2.647)	0.043	<0.001
	T3 vs. T1	4.817 (1.979, 12.121)	0.001		1.578 (0.613, 3.935)	0.304	
Model 4	T2 vs. T1	1.347 (0.649, 2.795)	0.423	<0.001	1.470 (0.899, 2.405)	0.124	<0.001
	T3 vs. T1	4.855 (1.950, 11.934)	0.001		1.544 (0.631, 3.780)	0.342	
**Frailty (Low physical activity)**							
Model 1	T2 vs. T1	1.947 (1.399, 2.711)	<0.001	<0.001	1.782 (1.388, 2.289)	<0.001	<0.001
	T3 vs. T1	3.300 (2.021, 5.390)	<0.001		3.713 (2.397, 5.753)	<0.001	
Model 2	T2 vs. T1	1.679 (1.206, 2.365)	0.002	<0.001	1.598 (1.290, 2.011)	<0.001	<0.001
	T3 vs. T1	3.323 (2.122, 5.396)	<0.001		3.206 (2.227, 4.995)	<0.001	
Model 3	T2 vs. T1	1.712 (1.328, 2.426)	0.002	<0.001	1.757 (1.357, 2.278)	<0.001	<0.001
	T3 vs. T1	3.257 (1.905, 5.601)	<0.001		3.084 (1.937, 4.932)	<0.001	
Model 4	T2 vs. T1	1.616 (1.150, 2.269)	0.006	<0.001	1.604 (1.237, 2.081)	<0.001	<0.001
	T3 vs. T1	2.994 (1.757, 5.102)	<0.001		3.154 (1.971, 5.047)	<0.001	
**Frailty (Low body weight)**							
Model 1	T2 vs. T1	1.935 (1.288, 2.906)	0.001	<0.001	1.864 (1.332, 2.608)	<0.001	<0.001
	T3 vs. T1	2.458 (1.240, 4.873)	0.010		1.355 (0.554, 3.313)	0.505	
Model 2	T2 vs. T1	1.975 (1.272, 3.075)	0.003	<0.001	0.839 (0.589, 1.234)	0.350	<0.001
	T3 vs. T1	2.227 (1.156, 4.467)	0.021		5.920 (2.364, 14.994)	<0.001	
Model 3	T2 vs. T1	1.729 (1.101, 2.715)	0.017	<0.001	0.837 (0.582, 1.204)	0.347	<0.001
	T3 vs. T1	1.563 (0.578, 3.718)	0.328		3.644 (1.297, 10.262)	0.011	
Model 4	T2 vs. T1	1.641 (1.035, 2.604)	0.035	<0.001	0.803 (0.557, 1.159)	0.242	<0.001
	T3 vs. T1	0.910 (0.421, 2.640)	0.910		2.934 (1.003, 8.587)	0.049	

a *Adjusted covariates:*

Model 1 = Unadjusted.

Model 2 = Model 1 + age, ethnicity, body mass index (BMI).

Model 3 = Model 2 + systolic blood pressure (SBP), serum fasting glucose, serum TG, serum creatinine.

Model 4 = Model 3 + history of congestive heart failure, stroke, diabetes mellitus, smoker, physical activity.

^b^Odds ratios were interpreted as change of frailty components for each increase in different proteinuria levels.

## Discussion

This study showed a statistically significant correlation between proteinuria and frailty. Proteinuria was strongly associated with every component of frailty in a Non-institutionalized, general population, even after adjustment for multiple covariates.

Frailty, conceptualized as a state of increased vulnerability to stress resulting from aging-related decline in physical function across multiple physiologic systems, predicts poorer outcomes in several medical specialties (10, 12, 13). Fried et al. proposed a phenotype to diagnose frailty (10). Another frailty measure is the frailty index, which operationalizes frailty as the fraction of 46 deficits present in an individual (11). The more health deficits an individual has, the frailer and more vulnerable the individual will be to adverse health outcomes. Research has described frailty from the perspective of people with chronic illnesses. Frailty is prevalent in patients with chronic obstructive pulmonary disease (COPD), and the strongest predictor of frailty among these patients was self-reported shortness of breath (14). The prevalence of frailty among those with CHF is high and presents a greater risk of adverse events, including hospitalization and mortality (15). Occasionally, in patients undergoing cardiac surgery, frailty is associated with an increased risk of morbidity, mortality, functional decline, and major adverse cardiac and cerebrovascular events (16). Elderly people with frailty who suffer from cancer are at an increased risk of chemotherapy intolerance, postoperative complications, and mortality (17). Thus, comprehensive geriatric assessment (CGA), a multidisciplinary diagnostic and treatment process that identifies psychosocial, functional, and medical limitations of a frail older person, is applied to develop individualized approaches toward cancer treatment (18). Patients with mild CKDs have a double risk of frailty, while moderate-to-severe CKD patients have an ~ 6-fold risk (6). Moreover, the influence of moderate-to-severe CKD on frailty exceeds that of other chronic illnesses, such as cancer, vascular disease, and other degenerative diseases (6).

Proteinuria is characterized by the presence of excess protein in the urine. The two mechanisms leading to proteinuria are: (1) the abnormal trans-glomerular passage of proteins due to changes in glomerular capillary permeability and (2) subsequent impaired reabsorption by the epithelial cells of the proximal tubuli (19). Urine dipsticks mainly detect albumin; however, light chains or other urine proteins can be missed even when they are present in significant quantities. The test becomes positive when proteinuria surpasses 15–30 mg/dL (300–500 mg/day, depending on urine volume) (20). Microalbuminuria is defined as an ACR of 30–300 mg/g, and macroalbuminuria is defined as ACR ≥ 300 mg/g (8, 9). High levels of urinary albumin excretion are associated with an increased all-cause mortality rate in the general population and high-risk patients, such as elderly subjects and those with hypertension, DM (21, 22). A community-based study conducted in Japan indicated that proteinuria is the most potent predictor of end-stage renal disease (ESRD), while the next most powerful predictor is hematuria (23). Furthermore, it can be a predictor of ESRD risk in all ethnic groups, including white, black, Hispanic, and Asian (24). Proteinuria is also implicated in CV mortality, risk of incident stroke, and atherosclerotic events. Proteinuria can be used as a marker to evaluate the therapeutic effects of CV medicine (25). Sarcopenic individuals have a higher proportion of albuminuria than those without sarcopenia after stratification based on the presence of hypertension, DM, MetS, and a higher homeostasis model assessment of insulin resistance (HOMA-IR) (26). Sarcopenia is defined as the involuntary loss of skeletal muscle mass that occurs with advancing age (27). Previous studies on sarcopenia included study populations that were community-dwelling (28–30) whereas frailty is more widely applied in institutionalized people. Therefore, this study evaluated the correlation between frailty and proteinuria among middle-aged and older people with MetS.

A consequence of MetS is endothelial dysfunction (31). The endothelium regulates the growth, tone, hemostasis, and inflammation in the circulation. Insults to the endothelium result in inflammation and endothelial dysfunction (32, 33). Proteinuria is a manifestation of endothelial dysfunction and inflammatory cell infiltration in the kidneys (34). A study indicated that frailty occurs at a high frequency among Pre-dialysis patients and is correlated with aging, obesity, and endothelial dysfunction (35).

Insulin resistance (IR) is one of the components of MetS. Podocytes attach to the basement membrane of the glomeruli and share a slit-pore membrane with each other, forming a filter for plasma water and solutes (36). Podocytes are insulin-sensitive cells, and the IR of podocytes may be related to cell death and contribute to proteinuria (37). DM increases the risk of frailty and is a leading cause of disability in older adults. MetS and IR are strong risk factors for DM and could lead to frailty (38).

Activation of the renin-angiotensin-aldosterone system (RAAS) is common in patients with MetS (39). The clinical use of angiotensin-converting enzyme inhibitors (ACEIs) or angiotensin II receptor blockers (ARBs) to improve proteinuria suggests that activated RAAS plays an important role in proteinuria (40). Emerging evidence confirms the role of RAAS in the activation of inflammatory pathways, which may lead to frailty (41).

From previous literature, one can speculate that MetS, proteinuria, and frailty are associated and share common pathological mechanisms. A cohort study found a 15% overall rate of frailty among people with elevated serum creatinine concentrations (42). Sarcopenia and frailty are two geriatric syndromes with partly overlapping phenotypes, and sarcopenia usually precedes frailty (43). A Korean survey suggested that the close relationship between sarcopenia and albuminuria may be due to mechanisms such as RAAS, inflammation, and IR (44). In a recent cross-sectional study, Yang et al. have reported the correlation between frailty and albuminuria in elderly Chinese inpatients through multiple regression analysis (45). Another study conducted by Chang et al. revealed that the degree of microalbuminuria relates to frailty in middle-aged and elderly individuals (7). The authors proposed that the pathophysiological link between albuminuria and frailty is attributed to shared CV risk factors. Taken together, the finding of our study may be related to various complex mechanisms, such as CV diseases, sarcopenia, IR and RAAS.

This study has several limitations. First, the cross-sectional design limited the conclusions regarding the causality between proteinuria and frailty. The association between proteinuria and frailty over time was not analyzed because these clinical variables were measured only once at enrollment. Second, the survey revealed an independent association between proteinuria and frailty but did not observe mortality or comorbidities. Third, the relationship between proteinuria and frailty may vary among different ethnic groups. Fourth, although the analyses were adjusted for potential confounding factors, some residual confounders cannot be ruled out. Fifth, the study utilized frailty phenotype to classify individuals as frail; however, there is still a debate on the conceptual and operational definition of frailty (46).

## Conclusion

In conclusion, the level of proteinuria has an independent positive correlation with the severity of frailty, and proteinuria affects all the components of frailty, including slow walking, weakness, exhaustion, low physical activity, and low body weight. For patients with proteinuria and metabolic syndrome, it is necessary to pay attention to the risk of frailty. For these high-risk groups, physical activity examination can detect frailty at an early stage, allowing to promptly provide relevant treatment.

## Data Availability Statement

The datasets presented in this study can be found in online repositories. The names of the repository/repositories and accession number(s) can be found in the article/Supplementary Material.

## Ethics Statement

The studies involving human participants were reviewed and approved by National Center for Health Statistics https://www.cdc.gov/nchs/nhanes/irba98.htm. The patients/participants provided their written informed consent to participate in this study.

## Author Contributions

P-KC: conceptualization, data curation, writing original draft, formal analysis, and methodology. Y-PC: writing original draft, investigation, project administration, resources, supervision, and validation. L-WW: conceptualization, data curation, methodology, formal analysis, writing review, and editing. All authors contributed to the article and approved the submitted version.

## Conflict of Interest

The authors declare that the research was conducted in the absence of any commercial or financial relationships that could be construed as a potential conflict of interest.

## Publisher's Note

All claims expressed in this article are solely those of the authors and do not necessarily represent those of their affiliated organizations, or those of the publisher, the editors and the reviewers. Any product that may be evaluated in this article, or claim that may be made by its manufacturer, is not guaranteed or endorsed by the publisher.
