# Supplementary material for: Proteinuria as a Nascent Predictor of Frailty Among People With Metabolic Syndrome: A Retrospective Observational Study
Source: Front Public Health. 2022 Mar 10;10:847533. doi: 10.3389/fpubh.2022.847533 (PMC8960196; doi:10.3389/fpubh.2022.847533)
Supplement: Supplementary file 1 [file Data_Sheet_1.docx]

Supplementary Material

**Supplementary Table 1. Association between frailty and proteinuria in male and female participants with metabolic syndrome (older ages, for example 65+).**

| **Models ^a^** | **Tertiles** | **Male group** | | | **Female group** | | |
| --- | --- | --- | --- | --- | --- | --- | --- |
|  |  | **Odds ratio**  **(95% CI)** | ***P***  **Value** | ***P***  **for Trend** | **Odds ratio**  **(95% CI)** | ***P***  **Value** | ***P***  **for Trend** |
| **Model 1** | T2 v.s. T1 | 3.472 (1.203, 10.022) | 0.021 | < 0.001 | 1.996 (1.344, 3.611) | 0.020 | < 0.001 |
|  | T3 v.s. T1 | 18.620 (6.790, 51.460) | < 0.001 |  | 4.062 (1.709, 9.652) | 0.002 |  |
| **Model 2** | T2 v.s. T1 | 3.170 (1.022, 8.197) | 0.034 | < 0.001 | 1.671 (0.932, 2.997) | 0.085 | < 0.001 |
|  | T3 v.s. T1 | 15.993 (5.756, 44.435) | < 0.001 |  | 4.178 (1.735, 10.062) | 0.001 |  |
| **Model 3** | T2 v.s. T1 | 3.417 (1.159, 10.075) | 0.026 | < 0.001 | 1.699(0.932, 3.097) | 0.084 | < 0.001 |
|  | T3 v.s. T1 | 15.121 (4.403, 51.927) | < 0.001 |  | 2.858 (1.012, 8.075) | 0.047 |  |
| **Model 4** | T2 v.s. T1 | 3.476 (1.157, 10.442) | 0.026 | < 0.001 | 1.649 (0.880, 3.090) | 0.118 | < 0.001 |
|  | T3 v.s. T1 | 14.810 (4.121, 53.223) | < 0.001 |  | 2.666 (0.848, 8.387) | 0.093 |  |

^a^ Adjusted covariates:

Model 1 = Unadjusted.

Model 2 = Model 1 + age, ethnicity, body mass index (BMI).

Model 3 = Model 2 + systolic blood pressure (SBP), serum fasting glucose, serum TG, serum creatinine.

Model 4 = Model 3 + history of congestive heart failure, stroke, diabetes mellitus, smoker, physical activity.

^b^ Odds ratios were interpreted as change of frailty for each increase in different proteinuria levels.

**Supplementary Table 2. Association between the frailty and proteinuria in participants with and without diabetes.**

| **Models ^a^** | **Tertiles** | **DM group** | | | **Non-DM group** | | |
| --- | --- | --- | --- | --- | --- | --- | --- |
|  |  | **Odds ratio**  **(95% CI)** | ***P***  **Value** | ***P***  **for Trend** | **Odds ratio**  **(95% CI)** | ***P***  **Value** | ***P***  **for Trend** |
| **Model 1** | T2 v.s. T1 | 2.423 (1.205, 4.872) | 0.013 | < 0.001 | 1.806 (1.344, 3.611) | 0.062 | < 0.001 |
|  | T3 v.s. T1 | 4.729 (2.172, 10.298) | < 0.001 |  | 7.286 (2.579, 20.580) | < 0.001 |  |
| **Model 2** | T2 v.s. T1 | 2.478 (1.022, 8.197) | 0.021 | < 0.001 | 1.496 (0.803, 2.789) | 0.205 | < 0.001 |
|  | T3 v.s. T1 | 5.041 (5.726, 41.940) | < 0.001 |  | 6.716 (2.355, 19.148) | < 0.001 |  |
| **Model 3** | T2 v.s. T1 | 2.960 (1.420, 6.167) | 0.004 | < 0.001 | 1.463 (0.771, 2.777) | 0.245 | < 0.001 |
|  | T3 v.s. T1 | 4.632 (1.800, 11.919) | 0.001 |  | 3.811 (1.080, 13.442) | 0.038 |  |
| **Model 4** | T2 v.s. T1 | 2.869 (1.279, 6.437) | 0.011 | < 0.001 | 1.491 (0.784, 2.836) | 0.223 | < 0.001 |
|  | T3 v.s. T1 | 4.061 (1.509, 10.928) | 0.006 |  | 4.631 (1.347, 15.920) | 0.015 |  |

^a^ Adjusted covariates:

Model 1 = Unadjusted.

Model 2 = Model 1 + age, ethnicity, body mass index (BMI).

Model 3 = Model 2 + systolic blood pressure (SBP), serum fasting glucose, serum TG, serum creatinine.

Model 4 = Model 3 + history of congestive heart failure, stroke, diabetes mellitus, smoker, physical activity.

^b^ Odds ratios were interpreted as change of frailty for each increase in different proteinuria levels.
